# Unraveling the Role of Heme in Neurodegeneration

**DOI:** 10.3389/fnins.2018.00712

**Published:** 2018-10-09

**Authors:** Deborah Chiabrando, Veronica Fiorito, Sara Petrillo, Emanuela Tolosano

**Affiliations:** Molecular Biotechnology Center, Department of Molecular Biotechnology and Health Sciences, University of Torino, Turin, Italy

**Keywords:** heme, neurodegeneration, FLVCR, FLVCR1, FLVCR1a, neuropathy

## Abstract

Heme (iron-protoporphyrin IX) is an essential co-factor involved in several biological processes, including neuronal survival and differentiation. Nevertheless, an excess of free-heme promotes oxidative stress and lipid peroxidation, thus leading to cell death. The toxic properties of heme in the brain have been extensively studied during intracerebral or subarachnoid hemorrhages. Recently, a growing number of neurodegenerative disorders have been associated to alterations of heme metabolism. Hence, the etiology of such diseases remains undefined. The aim of this review is to highlight the neuropathological role of heme and to discuss the major heme-regulated pathways that might be crucial for the survival of neuronal cells. The understanding of the molecular mechanisms linking heme to neurodegeneration will be important for therapeutic purposes.

## Introduction

Neurodegeneration is a complex process leading to the progressive and selective loss of neurons. Mitochondrial dysfunction, oxidative stress, protein aggregation and endoplasmic reticulum stress are well established pathways driving the neurodegenerative process (Lansbury and Lashuel, 2006; Lin and Beal, 2006; Xiang et al., 2017; Morris et al., 2018). Recent data suggest that iron and heme metabolism dysfunction may also play a crucial role. The involvement of iron in neurodegeneration has been well documented (Levi and Finazzi, 2014; Mena et al., 2015; Stockwell et al., 2017) and iron chelation has been proposed as a therapeutic option (Ward et al., 2015; Ashraf et al., 2018). Moreover, several neurodegenerative disorders have been associated to dysfunction of heme metabolism, thus supporting a crucial role for heme in the pathogenesis of such diseases.

The neurotoxicity of heme is evident during intracerebral or subarachnoid hemorrhages. In these pathological conditions, large amount of hemoglobin and heme are released in the brain promoting oxidative stress, lipid peroxidation, inflammatory response and finally cell death (Righy et al., 2016). To counteract the toxic effects exerted by hemoglobin and heme, human cells have evolved several detoxification mechanisms. During brain injury, the plasma proteins Haptoglobin and Hemopexin have proved to be protective against free hemoglobin and heme as primary scavenger systems (Hahl et al., 2013; Ma et al., 2016). Moreover, the induction of the heme-degrading enzyme heme oygenase-1 (HMOX1) in the brain has been reported in intracerebral and subarachnoid hemorrhage, and other neurodegenerative conditions (Panahian et al., 1999; Wang and Doré, 2007; Guan et al., 2009; Gozzelino, 2016).

Hemoglobin-derived heme is not the only form of heme that the nervous system can handle. Endogenously synthesized heme is also deleterious if not properly managed. Heme synthesis is an endogenous and essential process occurring in almost all tissues, including the nervous system (Smith et al., 2011; Gozzelino, 2016). Heme is endogenously synthesized to regulate a plethora of biological processes that may be particularly relevant in the nervous system. Among them, heme regulates energy production, ion channels, gene expression and microRNA processing (Smith et al., 2011). For these reasons, heme is essential for neuronal survival and differentiation. Recent data clearly indicate that the amount of intracellular heme available for regulatory functions (the so called “Heme Regulatory Pool” or “Labile Heme”) is determined by the balance between heme synthesis, catabolism, import and export (Chiabrando et al., 2014b; Reddi and Hamza, 2016). The identification of a growing number of neurodegenerative disorders due to mutations in genes involved in heme metabolism strongly support the idea that the control of labile heme is crucial to avoid neurodegeneration. How heme drives the neurodegenerative process is still incompletely understood. However, it is becoming clear that heme neurotoxicity could be only partially explained by the release of toxic iron.

Here, we provide an overview of heme-related neurodegenerative disorders. Moreover, we discuss the potential mechanisms leading to heme neurotoxicity. We believe that the understanding of these mechanisms will be essential in the future to identify novel therapeutic targets.

## Neurodegenerative disorders caused by heme metabolism dysfunctions

**FIGURE 1:**
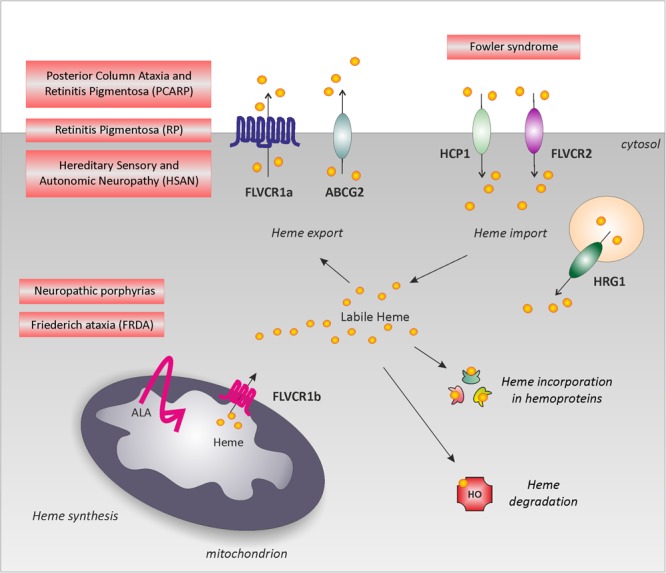
Neurodegenerative disorders associated to heme metabolism dysfunction. Schematic representation of the main pathways involved in the regulation of intracellular labile heme: heme synthesis, heme incorporation into hemoproteins, heme degradation, heme export and import. The picture highlights rare neurodegenerative disorders directly associated to heme metabolism dysfunction: defective heme biosynthesis causes neuropathic porphyria and is observed in FRDA; defective heme export (*FLVCR1* mutations) is responsible for PCARP, RP and HSAN. For simplicity, these disorders have been depicted near FLVCR1a; however, the specific contribution of FLVCR1a and FLVCR1b to these disorders still remains to be addressed. Finally, defective heme import (*FLVCR2* mutations) leads to the Fowler syndrome. Alteration of heme incorporation into hemoproteins and heme degradation have not been directly associated to a specific neurodegenerative disorder. However, we cannot exclude a role for these pathways in neurodegeneration.

The physiological relevance to control labile heme in the nervous system is highlighted by the identification of neurodegenerative disease-causing mutations in genes involved in heme metabolism. Indeed, mutations in genes involved in heme biosynthesis are responsible for neuropathic Porphyrias and Friederich Ataxia; mutations in the heme importer FLVCR2 have been found in Fowler Syndrome; mutations in the heme exporter FLVCR1 cause Posterior Column Ataxia and Retinitis Pigmentosa, non-syndromic Retinitis Pigmentosa and Hereditary Sensory and Autonomic Neuropathies (**Figure 1**). However, the molecular mechanisms underlying heme neurotoxicity are still unclear. Below, we summarize the major clinical features and the molecular genetics of these rare disorders.

### Neurodegenerative Disorders Due to Defects in Heme Synthesis

Heme synthesis is a well characterized and ubiquitous process that involves eight enzymatic steps. Briefly, ALAS1 (δ-aminolevulinic acid synthase 1) catalyzes the condensation of succynil-CoA and glycine in the mitochondrial matrix, to form δ-aminolevulinic acid (ALA). ALA is transported into the cytosol where it is converted to coproporphyrinogen III through a series of enzymatic reactions. Then, coproporphyrinogen III is translocated back into mitochondria for the final steps of heme synthesis (Chiabrando et al., 2014a). Mutations in each gene involved in the heme biosynthetic pathway are responsible for a group of rare disorders collectively named porphyrias (Besur et al., 2014; Ramanujam and Anderson, 2015). The partial deficiency of these enzymes results in reduced heme synthesis and accumulation of toxic porphyrin precursors in multiple organ systems, including the skin, liver and nervous system. Here we focus on neuropathic porphyrias which are associated with neurologic manifestations. Furthermore, defects in the last steps of heme synthesis have been reported in Friederic Ataxia (Schoenfeld et al., 2005; Huang et al., 2009).

#### Neuropathic Porphyrias

The most common neurologic manifestations in neuropathic Porphyrias are a combination of autonomic and peripheral neuropathy. The autonomic neuropathy is characterized by abdominal pain, tachycardia, hypertension, constipation and nausea. The peripheral neuropathy is predominantly a motor axonal neuropathy leading to muscle pain and weakness. Sensory neuropathy is manifested as neuropathic pain and distal paraesthesiaes. The central nervous system (CNS) may also be affected, leading to psychosis, anxiety, depression and seizures. Porphyric neuropathies are characterized by relative quiescent phases followed by acute neurovisceral attacks involving severe abdominal pain, peripheral neuropathies and psychiatric disturbances. Such attacks are often exacerbated by external stimuli (drugs and hormones) that induce endogenous heme synthesis thus leading to an increase of toxic heme precursors (Albers and Fink, 2004; Simon and Herkes, 2011; Tracy and Dyck, 2014). Neuropathic Porphyrias include delta-aminolevulinate dehydratase deficiency, acute intermittent porphyria, hereditary coproporphyria and variegate porphyria.

*Delta-aminolevulinate dehydratase deficiency* (ALAD deficiency; OMIM: #612740) is the only type of neuropathic porphyria with an autosomal recessive mode of inheritance. ALAD deficiency is an extremely rare disorder with childhood onset and severe neurologic implications. ALAD deficiency is caused by mutations in the gene encoding the second enzyme in the heme biosynthetic pathway. These mutations cause almost complete lack of ALAD activity and patients excrete a large amount of ALA into urine (Ramanujam and Anderson, 2015).

*Acute intermittent porphyria* (AIP; OMIM: #176000) is an autosomal dominant disorder with incomplete penetrance. It is the most common type of neuropathic porphyria. AIP is caused by mutations in the hydroxymethylbilane synthase (*HMBS*) gene, resulting in the partial deficiency of porphobilinogen deaminase (PBGD). PBGD is the third enzyme in the heme biosynthetic pathway and its reduced activity leads to the accumulation and urine excretion of ALA and porphobilinogen (PBG) (Pischik and Kauppinen, 2015; Ramanujam and Anderson, 2015).

*Hereditary coproporphyria* (HCP; OMIM: #121300) is an autosomal dominant disorder with incomplete penetrance. HCP results from mutations in the gene encoding coproporphyrinogen oxidase (*CPOX*). CPOX represents the sixth enzyme in the heme biosynthetic pathway. Its partial deficiency causes the accumulation of ALA, PBG and coproporphyrin III (Horner et al., 2013; Ramanujam and Anderson, 2015).

Variegate porphyria (VP; OMIM: #176200) is an autosomal dominant disorder with incomplete penetrance. VP is due to mutations in the gene encoding protoporphyrinogen oxidase (*PPOX*). PPOX catalyzes the oxidation of protoporphyrinogen IX to form protoporphyrin IX and its partial deficiency causes the elevation of plasma porphyrins (Horner et al., 2013; Ramanujam and Anderson, 2015).

The clinical presentation is generally milder in HCP and VP compared to AIP and ALAD deficiency.

The etiology of the neurological manifestations in neuropathic porphyrias remained undefined for long time. Nowadays, two major mechanisms have been proposed to contribute to the nerve failure: direct neurotoxicity of the porphyrin precursors and mitochondrial dysfunction (Lin et al., 2011).

#### Friederich Ataxia

Friederich Ataxia (FRDA; OMIM #229300) is a progressive neurodegenerative disease usually associated with cardiomyopathy and diabetes. Although a rare disorder, FRDA represents the most frequent type of inherited ataxia. FRDA is characterized by progressive gait and limb ataxia, dysarthria, areflexia, loss of vibratory and position sense, and a progressive motor weakness of central origin. Neurodegeneration first affects the dorsal root ganglia (DRG) but then involves spinal cord, peripheral nerves and cerebellum (Marmolino, 2011; Bürk, 2017).

FRDA is an autosomal recessive disorder caused by the abnormal expansion of the GAA triplet in the first intron of *FRATAXIN* (*FXN*) gene (Campuzano et al., 1996). FXN function remained elusive for long time. FXN is a mitochondrial iron chaperone involved in iron–sulfur (Fe–S) clusters and heme biosynthesis. Here we focus on the role of FXN on heme biosynthesis and readers are referred to more comprehensive reviews on the role of FXN in Fe–S clusters biosynthesis (Stemmler et al., 2010; Martelli and Puccio, 2014; Braymer and Lill, 2017; Rouault and Maio, 2017). FXN delivers iron to Ferrochelatase (FECH) (Yoon and Cowan, 2004), the enzyme responsible for the insertion of iron into protoporphyrin IX. Structural studies on the interaction between FXN and FECH shed light on the mechanism of iron delivery between them (Bencze et al., 2007; Söderberg et al., 2016). Evidences of defective heme biosynthesis have been reported in both cellular and mouse models of FXN deficiency as well as in FRDA patient-derived cells. Collectively these studies showed decreased expression of enzymes involved in the heme biosynthetic pathway (Schoenfeld et al., 2005; Huang et al., 2009), induction of HMOX1 (Huang et al., 2009), increased cellular protoporphyrin IX levels (Schoenfeld et al., 2005) and reduced heme content (Schoenfeld et al., 2005; Huang et al., 2009). Although the majority of these experiments have been performed in different cell and tissue types, it is conceivable that these findings can be translated to the nervous system. Fe-S clusters and heme biosynthesis are the two major iron consuming processes in mitochondria. Therefore, reduced iron utilization for Fe–S clusters and heme biosynthesis contributes to iron accumulation observed in FRDA (Huang et al., 2009). Moreover, mitochondrial iron accumulation in FRDA was reported to be due to heme-dependent upregulation of mitoferrin-2, a mitochondrial iron importer (Martelli et al., 2015). Both Fe–S clusters and heme are essential cofactors of the electron transport chain required for energy production. Indeed, FXN deficiency finally causes mitochondrial dysfunction, that actively contributes to the disease pathogenesis (Lodi et al., 1999; Martelli et al., 2015; Chiang et al., 2018).

Several yeast and mouse models of FXN deficiency have been generated (Perdomini et al., 2013). These models have been extremely useful to understand the molecular mechanisms of neurodegeneration associated to FXN loss and to test therapeutic approaches for the disorder.

### Neurodegenerative Disorders Due to Defects in Heme Import

The import of heme inside the cell is achieved through multiple transporters: the heme responsive gene 1 (HRG1), the Heme carrier protein 1/Proton-coupled folate transporter (HCP1/PCFT) and the Feline leukemia virus subgroup C receptor 2 (FLVCR2).

The role of HRG1 in the import of heme is well established (Rajagopal et al., 2008; White et al., 2013). HRG1 is highly expressed in the brain and its transient downregulation in zebrafish leads to hydrocephalus and yolk tube malformations suggesting an important role for HRG1 in neurodevelopment (Rajagopal et al., 2008). Conversely, the substrate-specificity of HCP1 and FLVCR2 have been debated. HCP1 was initially identified as a heme importer (Shayeghi et al., 2005). Nevertheless, Qiu A. et al. reported that HCP1 is a folate transporter implicated in hereditary folate malabsorption (Qiu et al., 2006). However, evidences suggest that the heme import function of HCP1 could be relevant in some cell types or in particular physiologic or pathologic situations (Chiabrando et al., 2014b). Because of the low expression level of HCP1 in the brain, the role of HCP1 in the import of heme in the nervous system is likely marginal.

FLVCR2 is a member of the major facilitator superfamily (MFS) of transporters (Pao et al., 1998; Law et al., 2008) initially proposed as a calcium-chelate transporter (Meyer et al., 2010). Subsequent studies revealed that FLVCR2 is an importer of heme (Duffy et al., 2010). FLVCR2 is ubiquitously expressed. In the nervous system, FLVCR2 is widely expressed throughout the brain and spinal cord (Duffy et al., 2010; Meyer et al., 2010). However, FLVCR2 expression and subcellular localization still remain to be investigated in detail due to the lack of a specific antibody. Among all these heme importers, *FLVCR2* is the only gene directly associated to a neurodegenerative disorder, the Fowler Syndrome. Therefore, the discussion below is focused exclusively on this disease.

#### Fowler Syndrome

Fowler syndrome, also known as Proliferative Vasculopathy, Hydrancephaly Hydrocephaly syndrome (PVHH; OMIM #225790), is a rare neurodegenerative disorder. The hallmark of the disease is the presence of proliferative glomerular vasculopathy in the CNS associated with severe hydrocephaly, ventriculomegaly, cortical thinning and hypoplastic cerebellum. Secondary features are hypokinesia and joint contractures (Meyer et al., 2010; Williams et al., 2010; Kvarnung et al., 2016). It is still unclear whether the proliferative vasculopathy is the primary or secondary event in the disease pathogenesis. Massive endothelial cells proliferation might be the first event leading to adjacent tissue damage, calcification and neuronal cells loss. Otherwise, proliferative vasculopathy might be a consequence of neurodegeneration (Meyer et al., 2010). The Fowler syndrome is characterized by early prenatal onset and is incompatible with life in most cases (Lalonde et al., 2010; Meyer et al., 2010; Thomas et al., 2010; Williams et al., 2010). Recently, two cases of survival beyond infancy have been reported. These patients were characterized by severe intellectual and neurologic disability (Kvarnung et al., 2016).

The Fowler syndrome is an autosomal recessive disorder caused by mutations in the *FLVCR2* gene (Meyer et al., 2010). Different kinds of mutations in the *FLVCR2* gene have been reported (Lalonde et al., 2010; Meyer et al., 2010; Thomas et al., 2010; Kvarnung et al., 2016), including missense and nonsense mutations, deletions and insertions. Homology modeling of FLVCR2 structure suggests that the missense mutations related with Fowler syndrome affect transmembrane domains that may modify the channel proper function or folding (Radio et al., 2018). It has been proposed that the mutations might lead to alteration of heme-iron metabolism. However, this is merely an hypothesis since *in vitro* or *in vivo* evidences are still lacking. The generation of appropriate *in vitro* and *in vivo* model systems will be important to investigate the molecular mechanisms underlying Fowler syndrome.

### Neurodegenerative Disorders Due to Defects in Heme Export

The export of heme is mediated by two widely expressed proteins: the ATP binding cassette subfamily G member 2 (ABCG2) and the Feline Leukemia Virus Subgroup C Receptor 1 (FLVCR1).

Besides heme, ABCG2 is involved in the transport of a variety of substrates: urate, chemotherapeutics, antibiotics, xenobiotics and food metabolites (Krishnamurthy and Schuetz, 2006). In the nervous system, ABCG2 is mainly located on the luminal side of endothelial cells (ECs) (Krishnamurthy and Schuetz, 2006), suggesting an important role for ABCG2 in the blood-brain-barrier to protect brain from drugs. Furthermore, ABCG2 has been implicated in brain protection following ischemic reperfusion injury (Shin et al., 2018) and in Alzheimer’s disease (Xiong et al., 2009; Zeng et al., 2012; Shen et al., 2010). However, the relevance of ABCG2-mediated heme efflux to neurodegeneration still remains to be elucidated.

Feline Leukemia Virus Subgroup C Receptor 1 (FLVCR1) is a member of the MFS of transporters (Pao et al., 1998; Law et al., 2008), implicated in the transport of heme and other planar porphyrins (Yang et al., 2010). *FLVCR1* gene encodes for two isoforms: FLVCR1a expressed at the plasma membrane and FLVCR1b in mitochondria (Chiabrando et al., 2012). FLVCR1 is ubiquitously expressed (Quigley et al., 2004; Keel et al., 2008; Chiabrando et al., 2012; Fiorito et al., 2014, 2015; Vinchi et al., 2014; Mercurio et al., 2015; Petrillo et al., 2017). In the nervous system, Flvcr1 mRNA has been detected in the mouse brain (neocortex, striatum, hippocampus, and cerebellum), posterior column of the spinal cord, retina and retinal pigment epithelium. The highest Flvcr1 mRNA levels have been found in the retina and spinal cord (Rajadhyaksha et al., 2010; Gnana-Prakasam et al., 2011). Unfortunately, no information is available concerning FLVCR1 protein expression levels and localization in neurons and glia, due to the lack of a reliable antibody against FLVCR1. Mutations in *FLVCR1* gene have been reported in distinct disorders affecting the sensory nervous system: Posterior Column Ataxia and Retinitis Pigmentosa, Retinitis Pigmentosa and Hereditary Sensory and Autonomic Neuropathy, as reviewed below.

#### Posterior Column Ataxia and Retinitis Pigmentosa (PCARP)

PCARP (OMIM: #609033) is a rare neurodegenerative syndrome characterized by sensory ataxia and retinitis pigmentosa. Sensory ataxia is a consequence of the degeneration of the posterior columns of the spinal cord, resulting in loss of proprioception. Retinitis pigmentosa is due to the progressive degeneration of photoreceptors in the retina, leading to night blindness and progressive restriction of the visual field (Higgins et al., 1997; Rajadhyaksha et al., 2010). PCARP has been described for the first time in Higgins et al. (1997) as an autosomal recessive disorder associated with the AXPC1 locus (Higgins et al., 1999). In 2010, the analysis of three inbreed families of different ethnic origins revealed that mutations in the *FLVCR1* gene were responsible for the disorder (Rajadhyaksha et al., 2010). In the majority of cases, PCARP is caused by homozygous mutations in the *FLVCR1* gene (c.361A > G – c.721G > A – c.574T > C – c.1477G < C) (Rajadhyaksha et al., 2010; Ishiura et al., 2011). The identified mutations are missense mutations that affect highly conserved residues in potential transmembrane domains of the heme exporter. *In vitro* studies suggest that these mutations result in the mislocalization of FLVCR1 protein and in the loss of its heme export function. Compound heterozygous mutations in the *FLVCR1* gene have been also reported in three siblings (c.1547G > A and c.1593 + 5_ + 8delGTAA) (Shaibani et al., 2015).

#### Retinitis Pigmentosa (RP)

Non-syndromic retinitis pigmentosa (RP; OMIM: #268000) encompasses a group of genetically heterogeneous disorders characterized by the progressive neurodegeneration of photoreceptors. RP-associated genes are involved in different processes: phototransduction, retinal metabolism, tissue development and maintenance, cellular structure and splicing (Hartong et al., 2006). Recently, *FLVCR1* mutations have been reported in some patients with retinitis pigmentosa (RP) without evidences of posterior column degeneration or cerebellar degeneration (Tiwari et al., 2016; Yusuf et al., 2018). Interestingly, all the patients carry a specific splice-site variant in *FLVCR1* (c.1092 + 5G > A) in homozygosity or as compound heterozygosity. Functional studies showed that the c.1092 + 5G > A mutation interferes with the correct splicing of *FLVCR1* resulting in the skipping of exon 4, the frame-shift deletion of 68bp and a premature stop codon (Tiwari et al., 2016; Yusuf et al., 2018).

#### Hereditary Sensory and Autonomic Neuropathy (HSAN)

HSANs are a group of neurodegenerative disorders of the peripheral nervous system mainly characterized by impaired nociception and autonomic dysfunction. The hallmark of the disease is the degeneration of sensory neurons, which transmit information about sensations such as pain, temperature and touch. The inability to experience pain causes unintentional self-injuries and chronic ulcerations. Soft tissue infections and osteomyelitis, often requiring amputations, are common. The disease is also associated to mild abnormalities of the autonomic nervous system: swallowing and feeding problems, apnea, gastro-esophageal reflux and delayed development are variable features of the disorder (Verpoorten et al., 2006; Rotthier et al., 2012; Auer-Grumbach, 2013).

HSANs arise from mutations in genes that are crucial for distinct molecular pathways: sphingolipid-metabolism, membrane-shaping of organelles, neurotrophin action, DNA methylation, regulation of ion channels, endoplasmic reticulum turnover and axonal trafficking and heme metabolism (Rotthier et al., 2012; Khaminets et al., 2015; Chiabrando et al., 2016; Tanaka et al., 2016). Recently, mutations in the *FLVCR1* gene have been reported in a subset of HSAN patients. Compound heterozygous mutations in *FLVCR1* (c.574T *>* C and c.610del – c.661C *>* T and c.1324dup) have been reported (Chiabrando et al., 2016). Functional studies in patient-derived cells showed that these mutations reduce heme export activity, enhance oxidative stress and increase the sensitivity to programmed cell death (Chiabrando et al., 2016). Finally, Castori et al. (2017) reported the homozygosity for a previously identified pc.661C > T variation in a sporadic case with a mixed phenotype of HSAN, PCARP and acute lymphocytic leukemia.

Due to the limited number of cases described to date, it is very difficult to understand whether FLVCR1-related PCARP, RP and HSAN are distinct disorders or rather a single clinical spectrum.

The finding of *FLVCR1* mutations in PCARP, RP and HSAN indicates that heme is particularly required for the survival of specific subpopulation of neurons. However, the elucidation of the role of heme in these subpopulations is far to be clearly understood.

The available mouse models of FLVCR1 deficiency did not recapitulate the major disease features of HSAN, PCARP or RP. The targeted disruption of *Flvcr1* gene causes embryonic lethality in two different mouse models. Mice lacking both Flvcr1a and Flvcr1b die *in utero* because of a block of erythropoiesis (Keel et al., 2008). Mice lacking only Flvcr1a die during embryonic development due to severe hemorrhages, edema and skeletal malformations (Chiabrando et al., 2012). The downregulation of FLVCR1 isoforms in zebrafish confirm the essential role of FLVCR1 isoforms in erythropoiesis and ECs maintenance (Mercurio et al., 2015).

The discrepancy between the phenotype of these animal models and the human diseases is likely due to different degree of Flvcr1a downregulation. Indeed, HSAN patients-derived cells still expressed FLVCR1a but are characterized by reduced heme export activity (Chiabrando et al., 2016). Moreover, treatment of patient-derived cells with Hemopexin, that facilitates heme export through FLVCR1a (Chiabrando et al., 2016), improved cell survival. These data clearly indicate that *FLVCR1* mutations do not completely abrogate FLVCR1a function (Chiabrando et al., 2016). Reasonably, embryonic lethality is expected for patients carrying null mutations in the *FLVCR1* gene.

The reason why the loss of an ubiquitously expressed heme exporter affects specific sensory modalities (vision, proprioception and/or nociception) is still unknown. The development of appropriate cellular and mouse models will help dissecting the role of FLVCR1 in these disorders.

## Heme-Regulated Processes Crucial for Neurourodegeneration

**FIGURE 2:**
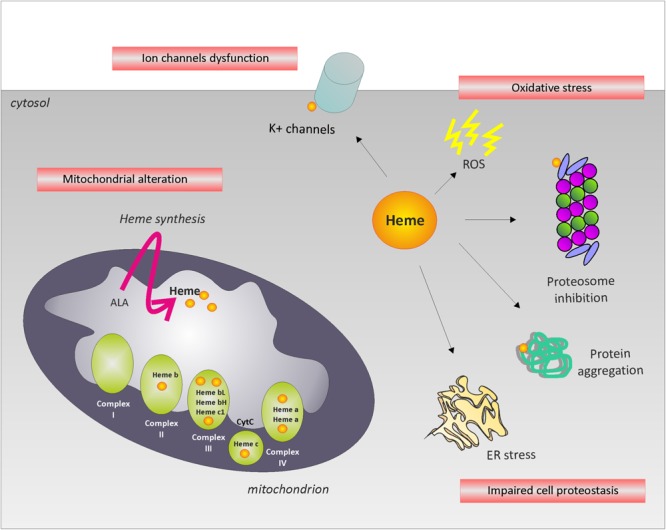
Potential mechanisms leading to neurodegeneration. The picture summarizes the major heme-regulated processes that might be relevant during neurodegeneration. The alteration of intracellular labile heme may affect cell proteostasis, oxidative phosphorylation, ion channels function and/or induce oxidative stress, leading to neurodegeneration.

In the paragraphs above, we summarized the most relevant neurodegenerative disorders associated to alterations in heme metabolism. However, the etiology of such diseases remains mostly undefined. Heme bears toxic properties due to its ability to promote iron mediated Fenton’s reaction. This awareness contributed over the years to underestimate the importance of iron-independent mechanisms of heme toxicity in neuronal diseases. The misconception that heme neurotoxicity is due to iron release from protoporphyrin IX mainly contributed to the lack of interest in the topic. To stimulate research in the field, we will discuss the major heme-regulated pathways that might be relevant during neurodegeneration (**Figure 2**).

### Heme and Oxidative Stress: Implications for Neurodegenerative Disorders

Oxidative stress is a condition occurring when the production of reactive oxygen species (ROS) overcomes the endogenous antioxidant defenses. Accumulation of ROS causes the oxidative damage of cellular components, included lipids, proteins and nucleic acids. As a pro-oxidant molecule, heme can itself promote oxidative stress, eventually leading to cell death. In particular, due to its hydrophobic nature, free heme tends to aggregate and bind to cell membranes. Oxidation of membrane components leads to increased permeability and altered lipid organization, in turn promoting cell lysis and death (Schmitt et al., 1993). The redox activity of heme-derived iron also contributes to the cytotoxic effects of heme. Indeed, in aqueous phase heme becomes unstable and releases free iron, responsible for the formation of ROS via the Fenton’s reaction (Kumar and Bandyopadhyay, 2005). The CNS is particularly susceptible to oxidative stress due to its high oxygen consumption rates and weak antioxidant systems. Consistently, oxidative stress is involved in the pathogenesis of several neurodegenerative disorders as Parkinson’s disease, Alzheimer’s disease, Huntington’s disease and FRDA (Schulz et al., 2000; Calabrese et al., 2005; de Vries et al., 2008; Li et al., 2013; Puspita et al., 2017). Increased ROS levels have been observed also in HSAN patient-derived cells (Chiabrando et al., 2016; Castori et al., 2017). To counteract the toxic effects exerted by ROS accumulation, human cells have evolved several detoxification mechanisms. In particular, the primary defense systems against ROS consists of oxidant-inactivating enzymes, such as superoxide dismutase or HMOX1, and endogenous antioxidants, including glutathione and thioredoxin (Li et al., 2013). The transcriptional response to oxidative stress is mediated by a *cis*-regulatory element known as ARE (antioxidant response element) found in the promoters of genes codifying for antioxidant proteins. Nuclear factor E2-related factor 2 (Nrf2) is the major trans-acting factor responsible for the activation of gene transcription through its binding to the AREs (Nguyen et al., 2009). Notably, alterations in Nrf2 signaling pathway have been found in Parkinson’s disease, Alzheimer’s disease, and Huntington’s disease (PMID: 18824091) as well as in a cardiac mouse model of FRDA (Anzovino et al., 2017). Several proteins and kinases modulate the Nrf2 pathway at various levels to ensure a tight control of its activity. Among these regulators, BTB domain and CNC homolog 1 (Bach1) acts as a repressor of Nrf2 activity by competing with Nrf2 for ARE binding sites. Notably, the activity and localization of Bach1 is regulated by heme (Sun et al., 2004; Suzuki et al., 2004; Marro et al., 2010), thus suggesting that alterations in heme homeostasis might affect the Nrf2-Bach1 axis and the cell ability to respond to the oxidative insult. This mechanism could be particularly relevant in the progression of neurodegenerative diseases, which is indeed exacerbated by oxidative stress. Hence, oxidative stress might exert neurotoxicity also by impairing the ubiquitin-proteasome system, as found in Parkinson’s disease (PD) where a decreased protein clearance rate due to elevated ROS results in the accumulation of alpha-synuclein (Hashimoto et al., 1999; McNaught and Jenner, 2001).

### The Role of Heme in Cellular Proteostasis: Implications for Neurodegenerative Disorders

The maintenance of protein homeostasis, also called proteostasis, is essential for cell function and survival (Hartl et al., 2011; Morimoto and Cuervo, 2014; Kaushik and Cuervo, 2015). The constant control of cellular proteostasis requires the proper activity of two main cellular components (Kaushik and Cuervo, 2015). On one hand, the activity of chaperones is fundamental to correct protein misfolding eventually occurring during protein synthesis, assembly in three-dimensional and functional structures and trafficking. On the other hand, protein clearance mechanisms mediate the removal of irreversibly misfolded proteins. In particular, this second purpose is achieved most prominently through two molecular systems: the ubiquitin-proteasome system (UPS) and the autophagy system. These systems are able to sense disturbances in the proteome and to be rapidly activated, thus exerting a constant protein quality control. In this way, the proteostasis network ensures the cell the ability to adapt to environmental changes and transitory stress. Reduced proteostasis capacity may lead to the accumulation of misfolded proteins, which form cytotoxic aggregates (Morimoto and Cuervo, 2014). Deficiencies in the machinery of protein homeostasis promote the onset and progression of several human pathologies, including neurodegenerative diseases (Hartl et al., 2011). For instance, deposits of aberrant proteins such as tau and α-synuclein, have been associated with dementia and Parkinson’s disease. The use of proteasome inhibitors as Bortezomib in cancer therapy has been shown to induce peripheral neuropathy as a prominent side effect (Alé et al., 2014; Kaplan et al., 2017). Moreover, an impairment of mitochondrial turnover via selective autophagy has been found in Parkinson’s disease (Andreux et al., 2013) and in sensory neuropathy (Khaminets et al., 2015). In addition to the above described classical proteostasis network, which assures the quality control of *de novo* synthetized cytosolic proteins, organelle-specific systems can be activated in response to proteotoxic stress occurring in specific subcellular compartments (Kaushik and Cuervo, 2015). These signaling pathways include the unfolded protein response (UPR) of the endoplasmic reticulum (ER) and mitochondria. In particular, the presence of unfolded proteins in the ER promotes the activation of three ER-resident transmembrane proteins: PERK (protein kinase RNA (PKR)-like ER kinase), ATF6 (activating transcription factor-6) and IRE1α (inositol-requiring enzyme-1α). The signaling cascade mediated by downstream effectors leads to an overall translational attenuation as well as enhanced transcription of ER chaperons. A similar mechanism has been described in mitochondria, which are particularly exposed to stressors that can trigger protein damage. Notably, alterations in the UPR machinery are involved in the pathogenesis of neurodegenerative diseases such as Alzheimer’s disease, Parkinson’s diseases, Huntington’s disease and prion disease, which are indeed characterized by accumulation and aggregation of misfolded proteins (Scheper and Hoozemans, 2015; Xiang et al., 2017). Finally, mutations in genes associated with ER function have been described in neurodegenerative disorders as Charcot-Marie-Tooth disease (CMT), hereditary neuropathies with proximal dominant involvement (HMSN-P) and amyotrophic lateral sclerosis (Beetz et al., 2013; Tsai et al., 2014; Yagi et al., 2016; Murakami et al., 2017).

Growing evidences indicate that the alteration of intracellular heme levels affects proteostasis. For instance, heme directly binds and inhibits the proteasome machinery promoting the formation of perinuclear “aggresomes” of ubiquitinated proteins (Vallelian et al., 2015). In particular, Vallelian et al. (2015) analyzed changes in protein expression upon heme treatment in mouse embryo fibroblasts (MEF) isolated from wild type and mice lacking HMOX1 (*Hmox1*-null mice). The study of the proteome in *Hmox1*-null MEF by mass spectrometry revealed a specific signature characterized by a strong upregulation of networks related to the response to unfolded proteins. These data suggest that excessive intracellular heme can trigger proteostasis disruption. In particular, heme overload correlates with accumulation of ubiquitinated proteins, thus indicating a reduced protein degradation capacity in heme-stressed cells (Vallelian et al., 2015). Consistent with these previous observations, the formation of protein aggregates has been described in macrophages during heme stress (Vasconcellos et al., 2016). In particular, cell treatment with increasing concentration of heme leads to the formation of aggresome-like induced structures (ALIS), which are characterized by the presence of p62/SQSTM1 (sequestosome-1) and ubiquitinated proteins. Importantly, the pre-treatment with ROS scavengers completely abolishes heme-induced ALIS formation in bone marrow-derived macrophages, thus indicating that ROS generation has a key role in this process (Vasconcellos et al., 2016). In addition, p62 is an inhibitor of the Nrf2 repressor Keap1 (Kelch-like ECH-associated protein 1). Therefore, the heme-induced sequestration of p62 in ALIS might affect the Nrf2-Keap1 axis (PMID: 24011591), interfering with the antioxidant response mediated by Nrf2-regulated genes. Enhanced ROS production and proteasome inhibition thus represent two different activities of heme, which turned out to be extremely cytotoxic in conditions of heme excess. These findings, taken together, point out a novel model of heme toxicity mediated by accumulation of damaged proteins and disruption of cellular proteostasis.

Another evidence of the proteotoxic effect mediated by heme comes from studies on ECs lacking FLVCR1a. The loss of the heme export way in ECs leads to the accumulation of endogenously synthetized heme, which in turn triggers programmed cell death via paraptosis and impairs the angiogenic process during embryonic development (Petrillo et al., 2017). Importantly, an altered swollen morphology of the ER as well as an increased expression of ER stress markers have been described in ECs isolated from embryos lacking *Flvcr1a* in endothelial cells (Petrillo et al., 2017). Interestingly, paraptosis has been described in neural development and neurodegeneration (e.g., amyotrophic lateral sclerosis and Huntington’s disease), in the ischemic damage and in the context of retina pathophysiology (Lee et al., 2016).

Although specific studies in neuronal cells are still lacking, we proposed that similar mechanisms might be relevant in the nervous system. Therefore, the disruption of cell proteostasis might be an important mechanisms contributing to neurodegeneration in conditions of altered intracellular heme levels.

### The Role of Heme in Mitochondria: Implications for Neurodegenerative Disorders

An additional mechanism through which heme could potentially participate to neurodegeneration is through the control of mitochondrial function. Mitochondrial dysfunction is a common pathogenetic mechanism of several neurodegenerative disorders. Particularly, the alteration of mitochondria by different mechanisms has been proposed as one of the underlying cause of Parkinson’s disease, Alzheimer’s disease, Huntington’s disease and amyotrophic lateral sclerosis (Shi et al., 2010; Costa and Scorrano, 2012; Bennett et al., 2014; Cabezas-Opazo et al., 2015; Arun et al., 2016; Bose and Beal, 2016).

Heme is a crucial cofactor for cytochromes *c* and cytochromes in complexes II, III and IV of the mitochondrial electron transport chain (ETC) (Kim et al., 2012), thus modulation of heme metabolism could interfere with oxidative phosphorylation. Moreover, some of the steps of heme synthesis occur in mitochondria and initiate by the condensation of succynil-CoA with glycine. Succynil-CoA is an intermediate of the TCA cycle, so heme production belongs to the group of TCA cycle cataplerotic pathways (Frezza et al., 2011; Fukuda et al., 2017), a set of processes whose function is to avoid the accumulation of TCA cycle metabolites. Alterations in heme biosynthesis, consequently, could impair this system thus altering mitochondrial function. In addition, heme has been reported to directly or indirectly influence adenosine triphosphate (ATP) translocation among mitochondria and cytosol (Sabová et al., 1993; Giraud et al., 1998; Azuma et al., 2008) and, therefore, modulation of heme homeostasis could profoundly affect the mitochondrial contribution to cell energy supply.

Supporting the role of heme in mitochondrial function, analyses on ECs demonstrated that alterations in heme metabolism, in addition to promote lipid peroxidation and activation of autophagy, induce mitophagy and apoptosis, indicating mitochondrial dysfunction (Higdon et al., 2012). Furthermore, we previously reported that loss of FLVCR1 impairs mitochondrial function in HeLa cells (Chiabrando et al., 2012) and mitochondrial morphology in human microvascular ECs (Petrillo et al., 2017).

Considering the role of heme in mitochondria, it is tempting to speculate that, by affecting mitochondrial function, the impairment of heme metabolism in neuronal cells could potentially initiate or sustain processes responsible for neuronal cells defects and death. Supporting this hypothesis, studies in the brain of HMBS-/- mice, a model of AIP, showed alterations in the activity of ETC complexes likely related to the compromised heme biosynthesis (Homedan et al., 2014). Moreover, it has been suggested that an energy failure may lead to a defective activity of the Na/K+ ATPase in the axon, leading to its degeneration (Lin et al., 2011). Since heme is required for ATP synthesis, we cannot exclude that heme metabolism could have a role in this context. Furthermore, defects in mitochondrial iron content, in the activity of the respiratory chain complexes and in ATP production have been observed in FRDA (Lodi et al., 1999). Although these defects in FRDA can be ascribed to alterations in Fe–S clusters trafficking, the contribution of heme to these processes cannot be excluded. Finally, in three cases of Fowler syndrome it was suggested the presence of a defect in complex III and IV of the ETC (Castro-Gago et al., 1999, 2001), directly indicating that mitochondrial defects due to alterations in heme trafficking could be the underlying cause of the neurological outcomes of this syndrome.

The contribution of mitochondrial dysfunction to PCARP, HSAN and RP has not been investigated. However, the implication of heme in these pathologies suggests the possibility that mitochondrial function could contribute to them. Notably, in sensory neurons the importance of heme in mitochondria could be related not only to the production of ATP, but also to mitochondrial dynamics (fusion, fission, motility). Mitochondrial dynamics are crucial for neurotransmission, synaptic maintenance and neuronal survival. Proper mitochondrial trafficking is particularly important in neurons as compared to other cell types due to their exceptional cellular morphology. Indeed, neurons extend their axons and dendrites for very long distances. Therefore, the neurons represent an extreme case of mitochondrial distribution: dysfunctions in mitochondrial distribution that are not dangerous for other cells, could be fatal for neuronal survival (Schwarz, 2013). Supporting this concept, compromised mitochondrial motility has been reported in Alzheimer’s disease (Cabezas-Opazo et al., 2015), Parkinson’s disease (Yang et al., 2008; Carelli et al., 2015), Huntington’s disease (Shirendeb et al., 2011) and in amyotrophic lateral sclerosis (De Vos et al., 2007; Shi et al., 2010).

In the case of human peripheral nerves or corticospinal tracts, axons can extend up to a meter, so the population of sensory neurons could be highly sensitive to defects in mitochondrial dynamics. Modulation of the cellular labile iron pool and mitochondrial iron content have been observed in FXN deficiency in association to alterations in both heme biosynthesis and OPA1 (mitochondrial dynamin like GTPase)-mediated mitochondrial fusion (Martelli et al., 2015). Although a causative link among these phenomena has not been proposed in FRDA, it is tempting to speculate that the modulation of iron and heme homeostasis could promote alterations in mitochondrial dynamics, with deleterious consequences in this pathology and, more likely, in neurological disorders affecting sensory neurons.

### Heme Regulates Ion Channels: Implications for Neurodegenerative Disorders

Emerging evidences suggest that labile heme is a signaling molecule that impact on the function of various ion channels involved in neuron excitability. Among them, *voltage-gated K+ channels* are a large family of K+ channels that open on membrane depolarization and contribute to maintain the resting potential and to determine the shape and frequency of action potentials in excitable cells (Sahoo et al., 2014). Heme directly binds to the *large-conductance calcium-dependent Slo1 BK channels* and *Kv1.4 A-type K+ channels*. The high affinity binding of heme to Slo1 BK channels decreases the frequency of channel opening leading to an inhibition of transmembrane K+ currents (Tang et al., 2003). Its binding to the N-terminal domain of the Kv1.4 channels inhibits the fast inactivation of the channel, thus reducing cellular excitability (Sahoo et al., 2013).

The activity of several K+ channels is modulated by carbon monoxide (CO) (Tang et al., 2004) and ROS (Sahoo et al., 2014; Peers and Boyle, 2015). Heme may also indirectly regulate the activity of several K+ channels through the generation of CO and ROS. Indeed, the degradation of heme by HO1 produces iron, biliverdin and CO. Moreover, the released iron is responsible for the formation of ROS via the Fenton’s reaction.

An alteration of the activity, function or expression of diverse K+ channels have been reported in Alzheimer’s disease, Parkinson’s disease, Huntington’s disease and ataxias (Peers and Boyle, 2015). As mentioned before, increased ROS is common to all these neurodegenerative diseases. Oxidative modulation of diverse K+ channels has been proposed as a pathogenetic mechansim leading to neurodegeneration. Finally, several studies highlight a crucial role of K+ channels in nociception (Busserolles et al., 2016). The identification of *FLVCR1* mutations in patients affected by HSAN (Chiabrando et al., 2016; Castori et al., 2017) suggests that the alteration of K+ channels activity may explain sensory defects in these patients. Considering all these data, it is possible to speculate that the alteration of labile heme may affect the activity of K+ channels, thus contributing to the neurodegenerative process.

## Concluding Remarks

The recent identification of several neurodegenerative disorders due to mutations in genes involved in heme metabolism highlights a previously unappreciated role for heme in neurodegeneration. Here, we discussed the potential mechanisms leading to heme neurotoxicity, thus suggesting future directions for investigation. The understanding of such mechanisms might be relevant for several neurodegenerative disorders. Indeed, alteration of heme metabolism have been observed also in Alzheimer’s disease, Parkinson’s disease and other aging-related neurodegenerative disorders (Atamna and Frey, 2004; Atamna, 2006; Scherzer et al., 2008; Dwyer et al., 2009; Smith et al., 2011; Hayden et al., 2015; Sampaio et al., 2017; Santiago and Potashkin, 2017; Fiorito et al., 2018). In the future, the comprehensive understanding of the molecular mechanisms linking heme to neurodegeneration will be potentially important for therapeutic purposes.

## Author Contributions

DC and VF conceptualized the study and wrote the manuscript. SP wrote the manuscript. ET wrote and reviewed the manuscript.

## Funding

This research was funded by University of Torino (Bando Ricerca Locale ex-60%).

## Conflict of Interest Statement

The authors declare that the research was conducted in the absence of any commercial or financial relationships that could be construed as a potential conflict of interest.
